# Appointments in Medical Schools

**Published:** 1858-11

**Authors:** 


APPOINTMENTS IN MEDICAL SCHOOLS.—Dr. John G. F. Holston has been appointed to the Chair of Surgery in the National Medical College at Washington, rendered vacant by the resignation of Professor May.

Dr. James B. McCaw, one of the editors of the *Virginia Medical Journal*, has been elected to the Chair of Chemistry in the Virginia Medical College, in place of Professor Martin Scott, resigned.

